# Penetrating Fish-Hook Ocular Injury: Management of an Unusual Intraocular Foreign Body

**DOI:** 10.1155/2014/901285

**Published:** 2014-04-13

**Authors:** Ludovico Iannetti, Paolo Tortorella

**Affiliations:** Department of Ophthalmology, “Sapienza” University of Rome, Viale del Policlinico 155, 00161 Rome, Italy

## Abstract

*Importance*. Ocular penetrating fish-hook injuries represent an unusual and very dangerous ocular trauma. We report the management of an unusual case of a simple-single barbed fish-hook accident globe injury successfully treated with surgery. *Observations*. We described a case report of a caucasian 32-year-old man presented with a scleral perforation of the left eye caused by a fish-hook injury while fishing. The fish-hook penetrated the sclera, passed the trabecular meshwork, and exited into the anterior chamber. He underwent surgery under local anesthesia to remove the intraocular foreign body and to repair the wound. The hook was removed backing through the entrance wound, enlarge the primary scleral laceration. Final visual outcome, one month after trauma, was 0.0 LogMar. *Conclusions and Relevance*. Our unusual case shows a modified extraction technique of fish-hook from the eye. Although the fish-hook injury represents generally a serious occurrence, in some cases, a prompt and appropriate method of extraction can lead to a good final outcome.

## 1. Introduction

Fish-hook injury is a really uncommon event and frequently occurs in the skin involving hands and fingers mostly. There are only a few cases of ocular penetrating fish-hook trauma published in the medical literature. All eye structures can be involved. Management of these injuries depends on type and location of the hook above all [1–3]. Aiello et al. reported four primary surgical techniques for the removal of fish-hooks embedded in nonocular tissue and their advantages and drawbacks in ophthalmic injuries. (a) The “back-out” or “retrograde” method, useful for barbless hooks, refers to backing the hook out through its entrance laceration. In globe trauma its use can bring excessive damage. (b) On the “snatch” or “string-yank” technique, not recommended for penetrating ocular injuries, downward pressure on the hook shank is used during a rapid extraction. (c) The “advance and cut” technique, the most useful method in anterior segment fish-hook laceration, consists of performing a controlled surgical incision to allow a traumatic release of the point and barb. The fish-hook is advanced through the tissue causing a second wound, the barb is then cut, and the remaining hook is backed out through the entry wound. (d) The “needle-cover” technique, the common choice for hook penetration of the retina, consists of passing a large bore needle into the eye through the hook entry laceration and then to engage the fish-hook barb within the lumen of the needle and both are removed at the same time [4, 5].

We report a case of penetrating simple-single barbed fish-hook injury for the unusual and unique penetration pattern never reported in ophthalmological literature. We used a modified surgical technique, different from the above mentioned methods, to remove the barbed fish-hook from the eye.

## 2. Observations

**Figure 1:**
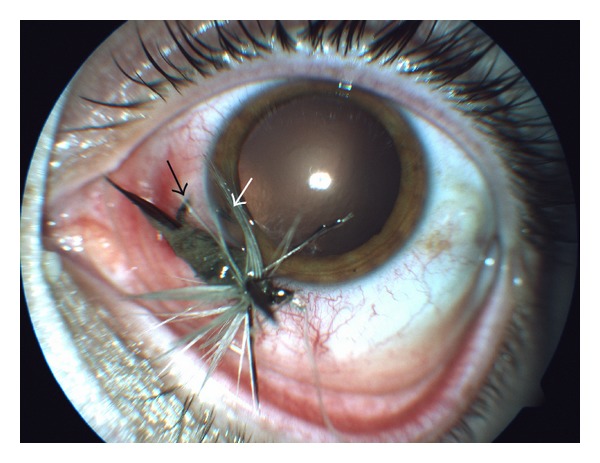
Photograph showing the barbed fish-hook penetrating the sclera exiting with its tip in anterior chamber. Pupil was in pharmacological mydriasis. (White arrow: fish-hook tip in the anterior chamber; black arrow: fish-hook through the scleral wound).

A healthy 32-years-old man was referred to our clinic with a fish-hook embedded in the left eye. The accident had occurred four hours earlier while the patient was fishing. On examination visual acuity (VA) was 0.20 LogMar. The foreign body, a simple-single barbed fish-hook, had penetrated the sclera 2 mm apart from the corneal limbus, passed through the trabecular meshwork, and exited in anterior chamber with only one entry wound. The barb was located in the trabecular meshwork; the hook point was visible in the shallow anterior chamber (Figure 1). No cornea, lens, or posterior segment damage was present.

**Figure 2:**
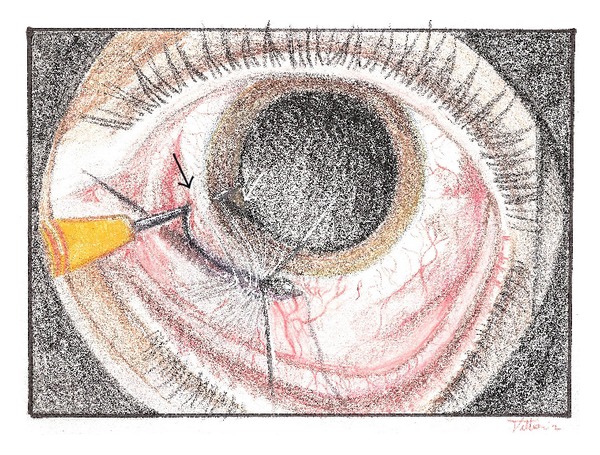
Drawing that shows the surgical technique used: the primary scleral laceration was enlarged surgically with a straight 15° blade to preserve the anterior chamber and to permit the fish-hook removal. (White arrow: fish-hook in the anterior chamber; black arrow: fish-hook tip through the scleral wound).

The patient underwent hook extraction the same day under local anesthesia. One mm wide incision was performed in clear cornea with a straight 15° blade to fill the anterior chamber with a viscoelastic substance. Conjunctiva was cut above the scleral wound. The fish hook was removed enlarging the entrance laceration with a straight 15° blade (Figure 2). The hook was pulled out backing through the entrance wound. Absorbable suture 7.0-vicryl was applied for scleral wound and conjunctiva. In agreement with patient tetanus prophylaxis was given in addition to systemic antibiotic therapy to prevent endophthalmitis.

**Figure 3:**
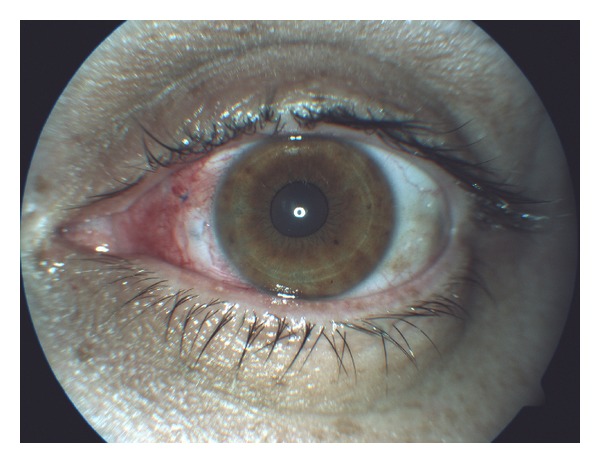
Photograph showing good eye conditions observed one week after the fish-hook surgical removal.

No postoperative complication was observed during the postoperative period. One week later VA was 0.0 LogMar and a good anatomical result was achieved (Figure 3). One month after surgery VA remained stable, and anterior segment and fundus oculi examination was normal. No IOP and retina abnormalities were observed during the follow-up.

## 3. Conclusions and Relevance

Management of penetrating ocular fish-hook injuries can be very difficult. Although these injuries often lead to serious consequences, in some cases, they may have a good long-term prognosis if prompt, appropriate surgical intervention is accomplished. Anterior segment damage is most commonly involved in penetrating fish-hook injuries, as entrance sites posterior to the limbus are only noted in 30% of cases and cornea is damaged mostly [4]. In our unusual case the barbed hook penetrated the sclera and the tip was lodged in anterior chamber with globe good preservation. To remove barbed hook we did not consider the surgical methods above mentioned for the globe injuries [4, 5]. We used an appropriate technique to remove the fish-hook and avoid major damage. To preserve corneal integrity we did not consider the “advance and cut” method, reported as the most useful technique for removing barbed hooks from the anterior segment. This safe surgical method is used to prevent the fact that the barb may engage the ocular tissue during removal of the hook [6]. Since only one penetrating wound was present, before removing the hook, we prefer to enlarge surgically the primary scleral laceration to preserve the anterior chamber. The hook was removed backing through the entrance wound. During the hook removal no further damage on ocular tissue was observed. Although resembling the “back-out” or “retrograde” method used for nonocular penetrating trauma, the technique we described is different because an enlargement of the original entrance wound was performed with a straight 15° blade in order to easily permit the retrograde exit of the fish hook. The present technique can be considered as a safe and new approach for such specific pattern of ocular penetrating fish-hook injury.
